# Aberrant expression of microRNAs 16 and 21 and gene targets in women with unexplained recurrent miscarriage: A case-control study

**Published:** 2018-10

**Authors:** Noorodin Karami, Seyed Hamidreza Mirabutalebi, Fatemeh Montazeri, Seyed Mehdi Kalantar, Mohammad Hasan Sheikhha, Maryam Eftekhar

**Affiliations:** 1 *Department of Genetics, Shahid Sadoughi University of Medical Sciences, Yazd, Iran.*; 2 *Abortion Research Centre, Yazd Reproductive Sciences Institute, Shahid Sadoughi University of Medical Science, Yazd, Iran. *; 3 *Research and Clinical Center for Infertility, Yazd Reproductive Sciences Institute, Shahid Sadoughi University of Medical Sciences, Yazd, Iran.*

## Introduction

Recurrent miscarriage (RM), as the loss of two or more pregnancies before the 20^th^ wk of gestation, is described by the American Society of Reproductive Medicine (1). Its prevalence in all pregnancies is about 1-5% (2) and can occur for different causes, including genetics, anatomical, infectious, hormonal, and immune deficiencies (3). However, the etiology of over 50% of miscarriage cases remains unexplained (4). 

Micro-RNAs, which were introduced for the first time in the early 2000s (5), are non-coding RNAs, that usually made up of 18-25 nucleotides, and regulate gene expression through degradation of target mRNA or blocking the translation of protein (6). Recently, it has been discovered that microRNAs play an important role in many diseases of the reproductive system, including endometriosis, preeclampsia, infertility, and RM (7). In 2012, a study demonstrated that microRNA polymorphisms (miR-146aC>G, miR-149T>C, miR-196a2T>C, and miR-499A>G) in Korean patients are associated with spontaneous abortion (8). Also, the results of an investigation confirmed the potential role of circulating microRNAs as a diagnostic biomarker in patients with unexplained spontaneous abortion (9).

Additionally, the lack of a complete understanding of the mechanisms associated with miscarriage is as a challenge to early detection of patients with RM and to discover the causes of the disease. One of these mechanisms can be a defect in the process of angiogenesis (10). Angiogenesis is the process of formation of new vessels from the pre-built vascular system (11). This process is a common feature of embryo implantation and tumor metastasis (12). Reducing the level of this process during pregnancy, can lead to abnormal growth of the fetus and miscarriage (13, 14). Even, new studies have shown that increased expression levels of genes involved in angiogenesis may lead to RM (15). The microRNAs 16 and 21 are the most important miRNAs involved in angiogenesis and miscarriage. In previous studies, it has been proven that one of the targets of miR-16 is vascular endothelial growth factor-A (*VEGF-A*), and is involved in the angiogenesis of the placenta. In embryonic villus sampling, the high expression level of this miRNA is associated with recurrent miscarriage (16). Also, phosphatase and tensin homolog (*PTEN*) is the main gene target of miR-21 which is indirectly involved in angiogenesis (17). 

The aim of this study was to evaluate the expression levels of microRNAs 16 and 21 and their gene targets in peripheral blood mononuclear cells (PBMCs) and plasma of women with unexplained RM (uRM) and controls.

## Materials and methods

**Sample collection**

In this case-control study, all participates referred to Yazd Research and Clinical Center for Infertility from September 2016 to March 2017 were evaluated in two groups: uRM group (n=25) and control group (n=25). The inclusion criteria for the uRM group were a history of at least two consecutive miscarriages before the 20^th^ wk of pregnancy, lack of autoimmune diseases, metabolic disorders, anatomical abnormalities, infections, and chromosomal disorders with parental origin. 

The control group included pregnant women with at least one healthy child and no history of pregnancy related disorders such as endometriosis, infertility, miscarriage, and the like. 5ml peripheral blood samples obtained from the participants (in the uRM group after miscarriage and in the control group before the elective pregnancy termination). Also, the age range for both groups was 18-40 yr. 

**Isolation of PBMCs and plasma**

The blood samples were collected using EDTA-tubes and centrifuged at 1200 g for 10 min at 25^o^C. The clear supernatant that was the plasma, isolated and transferred into a Ribonuclease/ deoxyribonuclease-free microtube. By using the Ficoll-Paque PLUS kit (GE Healthcare, USA), PBMCs were isolated and in the next step, washing and centrifuging was performed to remove platelets and extra plasma. The PBMCs and plasma samples were stored at -80^o^C until use.

**Total RNA extraction**

Total RNA was extracted from 200 µl plasma samples according to the manufacturer's instructions from the High Pure RNA Isolation Kit (Roche, Germany). Also, for the extraction of total RNA from PBMCs, PrimePrep blood RNA extraction kit (Genet Bio, Korea) was used. After extraction, to evaluate the quality of the samples, their concentration was measured by the NanoDrop Spectrophotometer. Then, the extracted samples from PBMCs were divided into two parts, one part for making the cDNA of the genes and another for the cDNA of microRNAs. 

**Real-time quantitative reverse-transcription polymerase chain reaction (PCR)**

In the present study, cDNA (complementary DNA) was synthesized using reverse transcription (RT) reaction. The BONmiR 1st-strand cDNA synthesis kit and miRNA-specific primers were purchased from Stemcellstech and microRNAs were converted to cDNA by the poly A method, which after the polyadenylation reaction, cDNA synthesis was performed. Each RT reaction consisted of 10 μl polyadenylated RNA, 0.8 μl dNTPs mix, 1 μl RT enzyme (50 U/ìL), 2 μl RT buffer, 5.2 μl Ribonuclease-free water, and 1 μl BON-RT primer, in a total volume of 20 μl. The reactions were carried out under the following conditions: 5 min at 55^o^C, 15 min at 25^o^C, 30 min at 42^o^C, and 5 min at 95^o^C. 

Also, for the synthesis of cDNA genes, High-Capacity cDNA Reverse Transcription Kit (Applied Biosystems, USA) was used according to its instructions which each reaction is as follows: 10 min at 25^o^C, 120 min at 37°C, 5 min at 85^o^C, and hold at 95^o^C.

The Applied Biosystems 7900HT Fast Real-Time PCR System was used to carry out real-time quantitative reverse transcription (qRT)-PCR. By using the High-Specificity miRNA quantitative PCR Core Reagent Kit BONmiR Kit (Stemcellstech, Iran), specific forward primer and universal revers primer, qRT-PCR was performed for the desired microRNAs. The specificity of each qRT-PCR reaction is determined by the forward primer.

The synthesized cDNAs in the previous step were subjected to qRT-PCR reactions using the following compounds: 1 μl cDNA, 0.5 μl miRNA-specific forward primer, 0.5 μl universal reverse primer, 6.5 μl miRNA quantitative PCR master mix and 4.5 μl Nuclease-free water, in a total volume of 13 μl. These compounds were mixed together and placed in the machine according to the following program: 95^o^C for 20 sec, followed by 40 cycles of 95^o^C for 5 sec and 60^o^C for 30 sec. Also, to perform the qRT-PCR reactions of the genes desired (not microRNAs), the RealQ Plus 2× Master Mix Green kit (Ampliqon, Denmark) was used according to the manufacturer's instructions.

**Ethical consideration**

The Ethics Committee of Shahid Sadoughi University of Medical Sciences, Yazd, Iran approved the protocol of the study (IR.SSU.MEDICINE.REC.1396.141). After receiving informed consent from all participants, blood samples were collected according to approved guidelines.

**Statistical analysis**

IBM SPSS Statistics software (Statistical Package for the Social Sciences, version 24.0, SPSS Inc, Chicago, Illinois, USA) and Graphpad Prism 6 (Graphpad Software) has been used to analyze the results of the study. Also, the Mann-Whitney U-test and *t*-test were used to compare the expression levels of the genes and miRNAs. Statistically, the significance level was considered as p<0.05.

## Results

The expression levels of microRNAs 16 and 21 in plasma were compared between the uRM group and the control group. Also, there were no significant differences in the age and body mass index between two studied groups (p=0.96 and 0.14, respectively). Additionally, the mean age of uRM and control groups was 27.52 and 27.48, respectively.

**Comparison of miR-16 and miR-21 expression levels in plasma**

**Figure 1:**
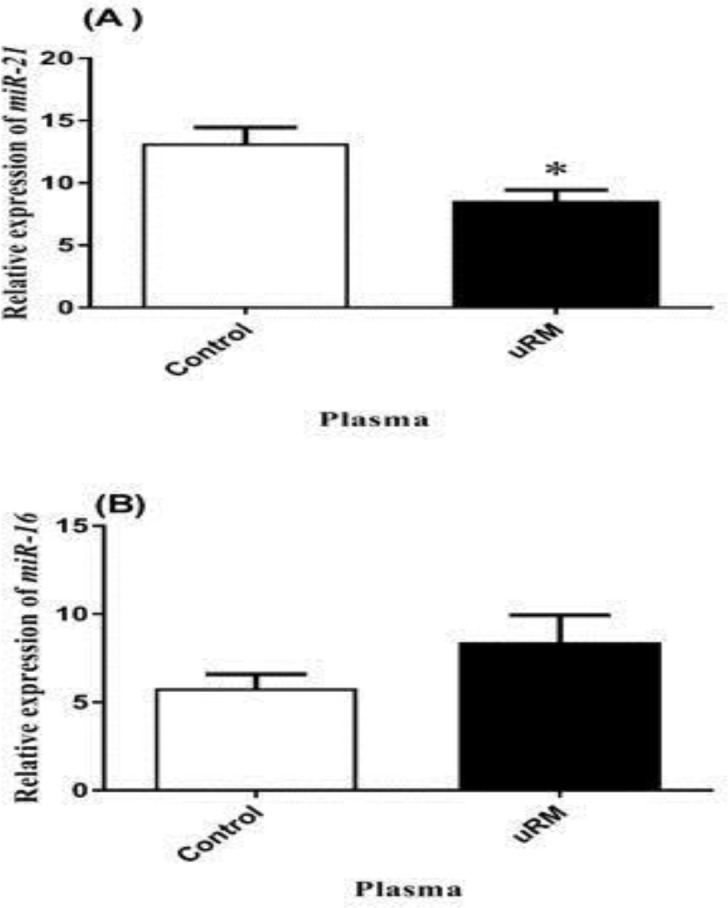
The relative expression of (A) miR-21 and (B) miR-16 in plasma. uRM: Unexplained recurrent miscarriage group, miR: miRNA.

The expression level of miR-21 in the uRM group was significantly lower than control group (p=0.04), however, no significant change was observed in the miR-16 expression (p=0.14) (Figure 1).

**Comparison of miR-16 and miR-21 expression levels in PBMCs**

**Figure 2:**
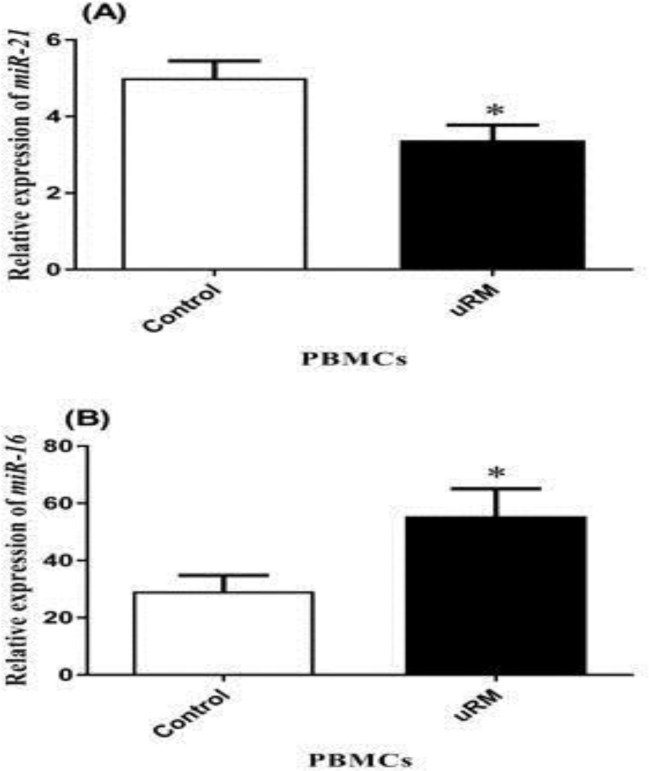
The relative expression of (A) miR-21 (p=0.02) and (B) miR-16 (p=0.04) in PBMCs.

The investigation of the expression of these microRNAs in PBMCs demonstrated that the expression of miR-16 and miR-21 in the uRM group compared to the control group were up-regulated and down-regulated, respectively (Figure 2).

**Comparison of** ***VEGF-A*** **and** ***PTEN*** **expression levels in PBMCs**

**Figure 3:**
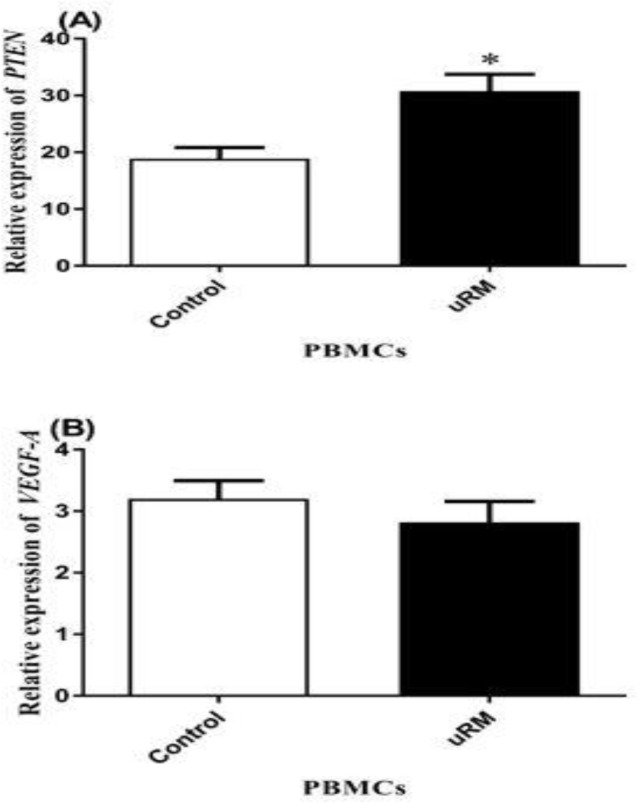
The relative expression of (A) *PTEN* and (B) *VEGF-A* in PBMCs.

The evaluation of the gene targets of these microRNAs in PBMCs showed that *PTEN* expression in the uRM group was up-regulated compared to the control group (p=0.03), while the expression of *VEGF-A* was relatively reduced in the uRM group, but this was not statistically significant (p=0.41) (Figure 3).

uRM: Unexplained recurrent miscarriage group

miR: miRNA

PBMCs: peripheral blood mononuclear cells

uRM: Unexplained recurrent miscarriage group

*PTEN*: phosphatase and tensin homolog

*VEGF-A*: vascular endothelial growth factor-A

PBMCs: peripheral blood mononuclear cells

## Discussion

In this study, we examined the expression of miR-16 and *VEGF-A* in both plasma and PBMCs and found higher expression level of miR-16 in the plasma of uRM group compared to the control group, but this was not significant. In PBMCs, its expression level was statistically significant. This was similar to results of Zhou and colleagues that showed the miR-16 expression was higher in the villi and decidua (16). Also, in these cells, the significance level of *VEGF-A* expression in the uRM group was more than 0.05. Thus, it can be concluded that there is no significant relationship between miR-16 and *VEGF-A* in mRNA levels. This result was incompatible with the study of Amirchaghmaghi and colleagues, which was carried out at the protein level (18). It is possible that this relationship exists only at the protein level.

In another investigation, we studied the expression of miR-21 and *PTEN* in PBMCs and showed that there is a correlation between their expression levels, so that miR-21 and *PTEN* in women with uRM were down-regulated and up-regulated, respectively. These findings were consistent with the study by Tokyol and colleagues, that showed a remarkable increase in the expression of *PTEN* in decidua and trophoblast cells of patients with RM (19). Also, plasma examination of these patients revealed the decreased expression of miR-21, which was similar to the findings of El-Shorafa and co-workers (20). 

Considering that it has been shown that embryonic development is associated with similar mechanisms with tumor invasion (21), there is a possibility for the involvement of these genes in feto-maternal angiogenesis. Several signaling pathways are associated with this process, including phosphatidylinositol-3-kinase (PI3K)/Akt and mammalian target of rapamycin (mTOR) (22). In 2011, a study conducted to examine the effect of miR-21 on human tumor cells demonstrated that with increasing the expression of this microRNA, extracellular-regulated kinase (ERK) and Akt signaling pathways are activated, and lead to increased expression of *HIF-1α* and *VEGF*, and also enhances angiogenesis (17). In RM, this process can occur inversely, which means that by reducing the angiogenesis, nutrient delivery to the fetus is reduced and miscarriage may take place (11). 

**Figure 4:**
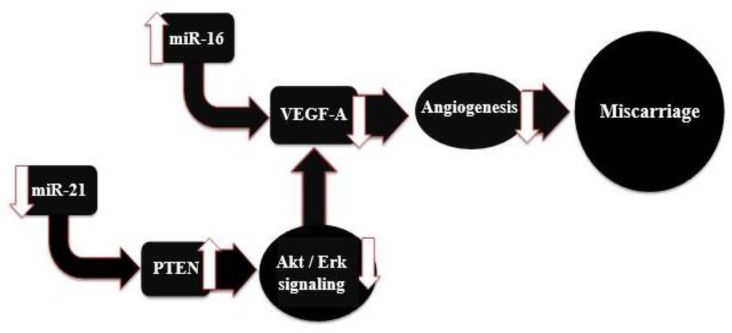
A proposed model for the relationship between miRNAs 16/21 and recurrent miscarriage

Although in our study, the *VEGF-A* expression was not significantly altered, in accordance with studies performed (18, 23), it is likely that its expression at the protein level will be significant. Therefore, further research is needed in this field and our proposed model for this study is shown in figure 4.

Finally, it's important to note that the lack of a non-invasive biomarker has always been one of the main challenges for patients with uRM; therefore, our study can open a window for more extensive research to determine a non-invasive biomarker for early diagnosis of these patients and to identify the causes of the disease.

## Conclusion

Our research is the first study that simultaneously examined the expression of both miRNAs 16 and 21 and their gene targets in the PBMCs of patients with uRM. Considering the inverse relationship between the under-expression of miR-21 and the over-expression of PTEN, and also the significant changes of this microRNA in both plasma and PBMCs, miR-21 plays an important role in the progression and etiology of RM. However, due to the relatively low population of patients in this study, more research is needed.

Therefore, qualitative and quantitative clinical trials in the future may determine the diagnostic potential of miR-21 as a specific circulating biomarker for women with uRM. Also, in this study, a number of major molecules of the angiogenic pathway, which are common between the invasion of cancer cells and embryonic development, were investigated in patients with uRM. The current study can be the pioneer of more extensive research in this field.

## Note

***This article extracted from the M.Sc. thesis. (Noorodin Karami)***
